# Early evaluation of a screen-and-treat strategy using high-risk HPV testing for Uganda: Implications for screening coverage and treatment

**DOI:** 10.7189/jogh.14.04157

**Published:** 2024-09-20

**Authors:** Marat Sultanov, Jurjen van der Schans, Jaap AR Koot, Marcel JW Greuter, Janine de Zeeuw, Carolyn Nakisige, Jogchum J Beltman, Marlieke de Fouw, Jelle Stekelenburg, Geertruida H de Bock

**Affiliations:** 1 Global Health Unit, Department of Health Sciences, University Medical Center Groningen, University of Groningen, Groningen, Netherlands; 2 Department of Economics, Econometrics and Finance, Faculty of Economics and Business, University of Groningen, Groningen, Netherlands; 3 Department of Radiology, University Medical Center Groningen, University of Groningen, Groningen, Netherlands; 4 Uganda Cancer Institute, Kampala, Uganda; 5 Department of Gynecology, Leiden University Medical Center, Leiden University, Leiden, Netherlands; 6 Department of Obstetrics and Gynecology, Medical Center Leeuwarden, Leeuwarden, Netherlands; 7 Department of Epidemiology, University Medical Center Groningen, University of Groningen, Groningen, Netherlands

## Abstract

**Background:**

Uganda has a high burden of cervical cancer and its current coverage of screening based on visual inspection with acetic acid (VIA) is low. High-risk HPV (hrHPV) testing is recommended by the World Health Organization as part of the global elimination strategy for cervical cancer. In this context, country-specific health economic evaluations can inform national-level decisions regarding implementation. We evaluated the recommended hrHPV screen-and-treat strategy to determine the minimum required levels of coverage and treatment adherence, as well as the maximum price level per test, for the strategy to be cost-effective in Uganda.

**Methods:**

We conducted a headroom analysis to estimate potential room for spending on implementing the hrHPV screen-and-treat strategy at different levels of coverage and treatment adherence (from 10% to 100%) at each screening round, and at different price levels of the hrHPV test. We compared the strategy with the existing VIA-based screen-and-treat policy in Uganda. We calculated headroom as the product of number of life years gained by the strategy and the willingness-to-pay threshold, minus the incremental costs incurred by the strategy. Positive headroom was interpreted as an indication of cost-effectiveness.

**Results:**

Compared with VIA-based screening with low 5% coverage, the hrHPV screen-and-treat strategy required at least 30% coverage and adherence for positive mean headroom, and compared with 30% VIA-based screening coverage, the minimum levels were 60%. At 60% coverage and adherence, the maximum acceptable price per hrHPV test was found to be between 15 and 30 international dollars.

**Conclusions:**

The hrHPV-based screen-and-treat strategy could be cost-effective in Uganda if the screening coverage and treatment adherence are at least 30% in each screening round, and if the price per test is set below 30 international dollars. The minimum required levels of screening coverage and adherence to treatment provide potential starting points for decision-makers in planning the rollout of hrHPV testing. The headroom estimates can guide the planning costs of screening infrastructure and campaigns to achieve the required coverage and treatment adherence in Uganda.

Cervical cancer is the fourth most common cancer in women globally, with an estimated 604 127 cases and 341 831 deaths in 2020 [1]. Low- and middle-income countries (LMICs) are disproportionately affected, accounting for more than 90% of cervical cancer mortality [2]. Compared to other cancers, the burden of cervical cancer has the largest inequity between high-income and low-income countries [3]. While this burden has been reduced in high-income countries through organised screening, LMICs have not achieved comparable reductions due to various factors, including resource constraints, inadequate infrastructure, and lack of expertise [4]. In sub-Saharan Africa, cervical cancer is the leading cause of cancer-related deaths among women [5]. In this regional context, Uganda represents an example of a country with a high burden of cervical cancer and inadequate screening, as it accounts for 25% of all cancer mortality, with an estimated 80% of cases presenting at a late stage [6,7]. The prevalence of high-risk HPV (hrHPV), the main cause of cervical cancer [2], is also high in Uganda, with estimates ranging between 10 and 40% among the female population [8,9]. While regular screening based on visual inspection with acetic acid (VIA) is officially recommended as part of the screening policy in Uganda [7], its implementation remains opportunistic in practice with low coverage rates, which are estimated to be well under 30% [10,11].

Considering the struggles of LMICs in establishing national cervical screening programmes, the World Health Organization (WHO) updated its guidelines for cervical cancer screening, recommending hrHPV testing as the primary screening method [12]. HrHPV testing has been estimated to be more sensitive for the detection of precancerous lesions than the conventional pap smear, as well as VIA, which is commonly used in LMICs [13]. Moreover, hrHPV testing can be implemented as a self-swab, which can potentially increase screening coverage, particularly among underscreened women [14–17]. To increase cervical cancer screening coverage in low-income countries such as Uganda, the WHO recommends a rapid transition to hrHPV-based screening from the existing VIA-based screening programmes [12].

While the WHO guidelines establish a clear framework for LMICs such as Uganda to adopt hrHPV testing, decisions regarding country-specific approaches to implementation require consideration of each country’s unique context. Model-based health economic evaluations, such as cost-effectiveness analysis, are usually performed to inform decision-makers regarding proposed health care interventions [18,19]. With increasing health care demand and costs, health economic evaluation methods are commonly used to inform resource allocation decisions in health care [20]. However, a full economic evaluation may not always be possible or necessary, particularly in the presence of high uncertainty regarding implementation, or because of a lack of detailed country-specific data for model input parameters. Both of these apply to the case of evaluating hrHPV-based cervical cancer screening in Uganda. In this context, early evaluation methods, such as headroom analysis, can provide insight into potential unmet clinical needs (i.e. achievable improvement in population health outcomes) and identify what is potentially needed to make the screening strategy cost-effective for a given country setting, leading to better-informed decision-making regarding implementation [21]. By identifying initial policy targets for key variables of the screening programme, specifically screening coverage and adherence to treatment of precancerous lesions, early evaluation methods can inform decision-makers in planning the national rollout of hrHPV-based screening.

In this study, we aimed to determine the minimum required levels of screening coverage and adherence to treatment of precancerous lesions for the hrHPV screen-and-treat strategy to be considered cost-effective in Uganda, and the maximum acceptable price of hrHPV test, through a model-based early evaluation approach.

## METHODS

We conducted this study in the context of the PREvention and SCReening Innovation Project Toward Elimination of Cervical Cancer (PRESCRIP-TEC), which investigated the feasibility of the recommended cervical cancer screening strategy using hrHPV self-testing in several countries, including Uganda, with a focus on underscreened populations [22].

In this study, we defined screening coverage as the proportion of eligible women who complete the primary screening test at a screening round. Since we did not model screening invitations separately for each woman, the screening coverage parameter in our study also represents screening uptake. Adherence to treatment of precancerous lesions was defined as the proportion of hrHPV-positive women who receive ablative or excisional treatment for precancerous lesions as part of the strategy. Screening rounds were defined as the ages at which eligible women are screened.

### Disease model

The SiMCerC model was originally constructed to evaluate the Dutch hrHPV screening policy, based on a microsimulation model of the natural history of cervical cancer (unpublished manuscript). We adapted this model for early evaluation in Uganda. The transition probabilities between disease states in the original model were independently informed by relevant literature and can be found in the Figure S1 in the **Online Supplementary Document**, which were retained in our model. We adjusted transition probabilities relevant to the country setting, related to the prevalence of hrHPV and cervical cancer survival. Using a lifetime horizon and yearly cycles, the model was used to simulate transitions between disease states for a cohort of 100 000 women in each model run. We implemented the model in the C++ programming language and compiled using XCode command-line tools, version 2397. Afterwards, we analysed and visualised the model output using R, version 4.2.2 (R Core Team, Vienna, Austria).

### Screening strategies

The strategy evaluated in our study is the screen-and-treat strategy with hrHPV self-sampling (hereinafter referred to as ‘main strategy’), recommended by the WHO [12] and implemented as part of PRESCRIP-TEC in Uganda. In line with the WHO guidelines, VIA is also part of this strategy as an assessment step to determine eligibility for ablative treatment (not to be confused with VIA as the primary screening test in the other strategies).

The existing VIA-based screening policy in Uganda is reported to be largely non-existent with low coverage rates, particularly among rural populations [7,10,23]. Considering the uncertainty in lifetime coverage rates in Uganda, reportedly between 4.8 and 30% [10], we evaluated the main strategy against two comparator strategies (**Table 1** ; Figures S3 and S4 in the **Online Supplementary Document**). The first is the existing VIA-based screen-and-treat strategy with low coverage (5%) (comparator 1), meant as a simplified representation of the business-as-usual scenario at the national level. The second comparator is the VIA-based screen-and-treat strategy with higher coverage (30%) and the same eligible ages and intervals as the main strategy (comparator 2). This strategy serves as an alternative to directly compare hrHPV testing with VIA as the primary screening test in the screen-and-treat approach.

**Table 1:** Screening strategies*

	Main strategy: hrHPV-based screen-and-treat	Comparator 1: VIA-based screen-and-treat (current policy)	Comparator 2: VIA-based screen-and-treat with higher coverage
**Screening test**	hrHPV test	VIA	VIA
**Range of eligible ages in years†**	30–50	25–49	30–50
**Interval in years**	5	3	5
**Follow-up in years**	1	1	1
**Screening coverage, %‡**	10–100	5	30
**Adherence to treatment of precancerous lesions, %§**	10–100	84 [24]	84 [24]

hrHPV - high-risk human papillomavirus, VIA - visual inspection with acetic acid

*The population was a hypothetical cohort of 100 000 women; individually simulated from age 0 until death. It was subject to the same all-cause mortality [25] and the same hrHPV prevalence [26].

†Eligible ages, representing screening rounds, are set by the WHO guidelines (starting from 30 with five-year intervals) and the existing Uganda policy (ending at 50). Comparator 1 is set to start from age 25 following the existing Uganda policy.

‡Both screening coverage and treatment adherence apply at each screening round in the model. Screening coverage per screening round in our model is equivalent to uptake of screening, since all eligible women at each round are considered. The model does not include invitations to screening as part of the screening flow.

§We assumed a high treatment adherence level for the comparator strategies based on previously reported project results, which may not be reflected in practice for these strategies. The parameter thus serves as an optimistic assumption for the comparators.

### Model parameters

We conducted a targeted literature search to identify relevant parameters for the Ugandan setting, including adjustments to the parameters of the disease model and the screening-related parameters for the modelled strategies (Table S2 in the **Online Supplementary Document**). Given the limited evidence available for the setting, the search focussed on previous economic evaluations of cervical cancer screening in Uganda, studies on the implementation of cervical cancer screening (including but not limited to hrHPV testing) in Uganda and Sub-Saharan Africa, age-specific hrHPV prevalence in Uganda, and cervical cancer survival rates in the region. The search approach was informed by existing recommendations for evidence identification [27]. For cost parameters, we adopted the health system (payer) perspective, representing only direct medical costs in the model (i.e. screening-related costs, costs of treatment of precancerous lesions, and cancer treatment costs). All costs were converted to 2022 international dollars (I$) [28].

### Model validation

The model parameters were validated in consultation with subject experts involved in the PRESCRIP-TEC project. For our adaptation, we retained the original transition probabilities for the progression and regression of pre-cancer and cervical cancer, focussing on adjusting the country-specific parameters related to hrHPV infection and cervical cancer survival. This was considered an acceptable assumption for the purposes of an early evaluation model. We calibrated the transition probabilities from hrHPV-negative to hrHPV-positive state to fit reported age-specific hrHPV prevalence rates in Uganda [26] (Figure S2 and Table S1 in the **Online Supplementary Document**).

### Statistical analysis

We first simulated the main strategy at fixed 100% coverage to explore the effectiveness gap, representing the maximum achievable improvement resulting from the strategy. We performed a total of 100 simulations, recording life years gained per woman and incremental costs per woman for each simulation.

#### Headroom analysis: Screening coverage and treatment adherence

To estimate the headroom at different levels of screening coverage and treatment adherence, we adjusted these parameters in 10% increments, ranging from 10% to 100%, resulting in a total of 100 combinations. We conducted 20 simulations for each combination, considering both the computation time and the relatively small increase in variability with higher numbers of simulations. For each of the 100 combinations, we estimated the headroom for each of the 20 simulations comparing the main strategy against comparator 1 and comparator 2. We identified the minimum required levels for a positive headroom, indicating cost-effectiveness in this early evaluation, using the mean headroom estimates across 20 simulations of each combination.

We calculated the headroom by multiplying life years gained per woman by the willingness-to-pay (WTP) threshold, and subtracting incremental costs per woman:

*Headroom* (*I*$) = (*Life years gained per woman* × *WTP* (*I*$)) − *Incremental cost per woman* (*I*$)

In the context of health economic evaluation, the WTP represents an upper limit to spending to gain an additional life year in a country’s health care system. Considering the limited resource availability for the health care system in Uganda, with the health expenditure accounting for less than 4% of the country’s gross domestic product (GDP) [29], we chose to adopt a strict WTP of 0.1 GDP per capita per life year gained, instead of commonly used thresholds of 1 to 3 GDP per capita.

Additionally, to show how the WTP parameter uncertainty impacts the potential cost-effectiveness of the evaluated strategy, we repeated the simulation 100 times using the identified combination of minimum screening coverage and treatment adherence levels and calculated the headroom for each simulation for a range of WTP thresholds. The proportion of simulations with positive headroom was calculated for WTP thresholds from 0.05 to 1 times GDP per capita, representing the probability of ‘acceptability’ of the main strategy under different WTP thresholds.

#### Headroom analysis: hrHPV test price

Using the identified minimum levels of screening coverage and treatment adherence, we varied the hrHPV test price parameter from I$ 5 to I$ 40 to determine the maximum acceptable price per test. In our model, these prices represent the cost of the test per woman screened during a single screening round, not the total lifetime screening costs.

## RESULTS

### Effectiveness gap: Maximum achievable improvement

**Figure 1:**
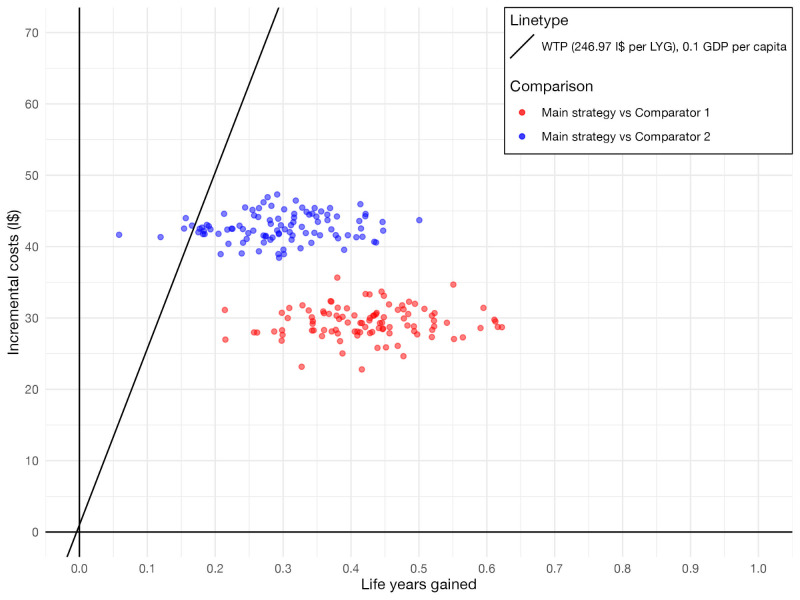
Life years gained and incremental costs of the main strategy at 100% coverage. The main strategy is simulated with 100% coverage and the same adherence as the comparators. Each simulation (100 in total) is represented by two points, one for each comparison (see figure key). The diagonal line represents the WTP, whereby the results under the line are interpreted as cost-effective. GDP – gross domestic product, I$ – international dollar, LYG – life year gained, WTP – willingess-to-pay.

The results of 100 simulations of the main strategy with 100% screening coverage are presented on a cost-effectiveness plane (**Figure 1**). Across 100 simulations, the mean life years gained per woman were 0.425 (standard deviation (SD) = 0.087) for the main strategy vs comparator 1, and 0.295 (SD = 0.081) vs comparator 2. These estimates represent the maximum achievable health improvement under perfect coverage of the main strategy for the entire eligible female population of Uganda, which can be interpreted as an average increase in female life expectancy.

The corresponding mean incremental costs per woman in the main strategy were I$ 29.37 (SD = 2.19) vs comparator 1 and I$ 42.68 (SD = 1.93) vs comparator 2. These costs represent the average incremental lifetime costs of the main strategy per woman in the population for each comparison, not the costs per woman screened. The increase in incremental costs between the comparisons represents the additional costs associated with preventing more cancer cases. By preventing fewer additional cancer cases vs comparator 2 than vs comparator 1, the main strategy accumulated higher lifetime average costs in that comparison. In 5% of the simulations, the WTP threshold was exceeded in the comparison between the main strategy and comparator 2, while in the other comparison, all simulations remained below the threshold.

### Headroom analysis: Minimum required coverage and treatment adherence

We determined the mean headroom estimates across 20 simulations for each screening coverage and treatment adherence level. The minimum levels required for positive mean headroom were 30% coverage and 30% adherence vs comparator 1 (**Table 2**), and 60% coverage and 60% adherence vs comparator 2 (**Table 3**). To simplify the comparison, we proceeded with the 60% coverage and 60% adherence levels for the subsequent analysis. The large variation in these results stems from smaller variations in life years gained (Table S3 in the **Online Supplementary Document**) multiplied by WTP to estimate the headroom.

**Table 2:** Headroom vs comparator 1, mean I$ (SD)

	Adherence to treatment in %									
**Coverage (%)**	**100**	**90**	**80**	**70**	**60**	**50**	**40**	30*	**20**	**10**
**100**	78 (16.23)	71.43 (23.43)	67.56 (16.95)	65.66 (20.09)	59.21 (23.75)	52.88 (21.85)	39.99 (22.13)	27.57 (18.01)†	1.26 (23.6)	−32.75 (22.54)‡
**90**	77.14 (16.38)	76.49 (25.7)	65.43 (23.21)	67.52 (19.09)	64.59 (16.93)	57.44 (22.32)	42.97 (18.79)	31.75 (15.69)†	1.51 (20.12)	−41.77 (23.45)‡
**80**	74.65 (23.52)	77.51 (18.32)	67.7 (16.91)	66.72 (16.61)	62.86 (16.24)	51.19 (15.81)	31.79 (17.65)	28.72 (23.57)†	6.61 (18.19)	−30.47 (20.29)‡
**70**	72.26 (13.23)	70.87 (18.56)	72 (14.7)	63.19 (24.1)	54.2 (23.71)	42.32 (20.26)	39.96 (24.78)	21.12 (27.12)†	1.4 (22.83)	−30.94 (23.62)‡
**60**	78.13 (16.82)	64.72 (13.39)	63.35 (16.73)	65.51 (15.08)	53.84 (17.76)	48.55 (21.67)	31.69 (18.59)	20.14 (17.34)†	−3.5 (22.93)‡	−29.8 (18.97)‡
**50**	60.57 (21.56)	57.63 (18.09)	53.11 (26.73)	55.7 (21.5)	44.4 (19.2)	36.91 (22.34)	22.19 (14.95)	0.23 (20.42)†	−8.99 (23.45)‡	−28.51 (20)‡
**40**	56.05 (18.43)	51.2 (22.8)	51.87 (21.17)	45.33 (22.89)	33.09 (23.7)	31.89 (18.69)	24.37 (22.35)	10.41 (19.33)†	−13.11 (19.14)‡	−26.64 (18.24)‡
30*	46.01 (22.34)†	40.11 (18.96)†	39.28 (22.6)†	29 (20.62)†	25.86 (19.53)†	14 (23.68)†	1.45 (20.14)†	0.45 (20.08)†	−1.91 (25.49)‡	−23.68 (19.91)‡
**20**	20.89 (23.29)	20.3 (22.28)	26.88 (21.38)	14.74 (22.87)	9.7 (18.34)	3.55 (24.76)	6.31 (17.65)	−4.8 (20.74)‡	−25.16 (25.16)‡	−18.07 (16.6)‡
**10**	11.08 (21.52)	3.38 (23.85)	−2.58 (15.09)‡	1.06 (20.68)	−6.39 (20.5)‡	−13 (23.45)‡	−17.31 (20.27)‡	−22.98 (18.44)‡	−16.86 (23.97)‡	−22.53 (23.67)‡

*Minimum required levels.

†Negative headroom results. Interpreted as levels at which the main strategy was not cost-effective vs comparator 1 on average.

‡The minimum levels were identified as the levels at which coverage and adherence were both the lowest. Thus, we chose 30%/30% in favour of 20%/40%.

**Table 3:** Headroom vs comparator 2, mean I$ (SD)

		Adherence to treatment in %									
**Coverage (%)**		**100**	**90**	**80**	**70**	60*	**50**	**40**	**30**	**20**	**10**
**100**		30.13 (14.54)	27.25 (21.48)	18.76 (15.08)	21.23 (21.04)	10.11 (20.63)†	2.4 (16.29)	−7.44 (19.63)‡	−14.47 (24.83)‡	−42.47 (18.12)‡	−84.44 (18.51)‡
**90**		30.36 (19.23)	33.89 (27.17)	17.85 (23.19)	18.62 (20.97)	14.55 (19.51)†	14.74 (15.47)	−0.88 (18.21)‡	−23.81 (23.26)‡	−42.96 (17.53)‡	−82.72 (22.05)‡
**80**		30.47 (18.26)	34.88 (18.95)	25.38 (24.39)	16.78 (14.79)	12.16 (21.23)†	2.84 (24.53)	−7.53 (25.07)‡	−18.28 (24.2)‡	−35.86 (23.97)‡	−77.56 (25.54)‡
**70**		25.62 (21.09)	22.48 (21.93)	28.35 (20.84)	14.61 (14.19)	12.47 (21.47)†	4.63 (23.81)	−3.87 (23.29)‡	−23.35 (22.29)‡	−43.86 (22.12)‡	−84.06 (23.93)‡
60*		28.94 (16.03)†	12.64 (19.86)†	19.38 (22.42)†	15.28 (19.57)†	9.06 (19.29)†	−0.38 (17.29)‡	−12.26 (21.89)‡	−29.25 (14.92)‡	−45.6 (25.22)‡	−76.49 (19.53)‡
**50**		16.97 (24.26)	16.03 (18.58)	12.08 (23.51)	7.76 (18.76)	−2.94 (14.87)‡	−11.59 (23.7)‡	−20.66 (18.8)‡	−36.6 (25.45)‡	−52.55 (23.84)‡	−79.39 (16.58)‡
**40**		15.12 (18.53)	4.5 (20.04)	3.83 (23.66)	−4.08 (24.29)‡	−14.38 (22.27)‡	−18.23 (19.51)‡	−17.61 (19.48)‡	−45.27 (19.62)‡	−62.77 (19.72)‡	−68.41 (19)‡
**30**		−9.45 (20.72)‡	−4.54 (20.19)‡	−9.94 (21.78)‡	−16.22 (25.5)‡	−19.17 (21.9)‡	−32.75 (25.31)‡	−40.38 (21.53)‡	−42.37 (19.46)‡	−60.12 (17.92)‡	−69.16 (23.41)‡
**20**		−26.37 (22.32)‡	−25.7 (20.43)‡	−8.72 (21.28)‡	−31.25 (23.23)‡	−38.38 (23.44)‡	−42.73 (19.2)‡	−45.82 (24.56)‡	−54.17 (22.25)‡	−69.11 (23.43)‡	−72.5 (19.95)‡
**10**		−41.72 (16.74)‡	−49.6 (23.56)‡	−46.31 (20.01)‡	−40.33 (22.48)‡	−52.26 (24.92)‡	−59.23 (20.18)‡	−66.45 (25.9)‡	−70.61 (22.27)‡	−64.42 (23.55)‡	−73.41 (25.07)‡

*The minimum levels were identified as the levels at which coverage and adherence were both the lowest. Thus, we chose 60%/60% in favour of 50%/70% or 70%/50%).

†Minimum required levels.

‡Negative headroom results. Interpreted as levels at which the main strategy was not cost-effective vs comparator 2 on average.

**Figure 2:**
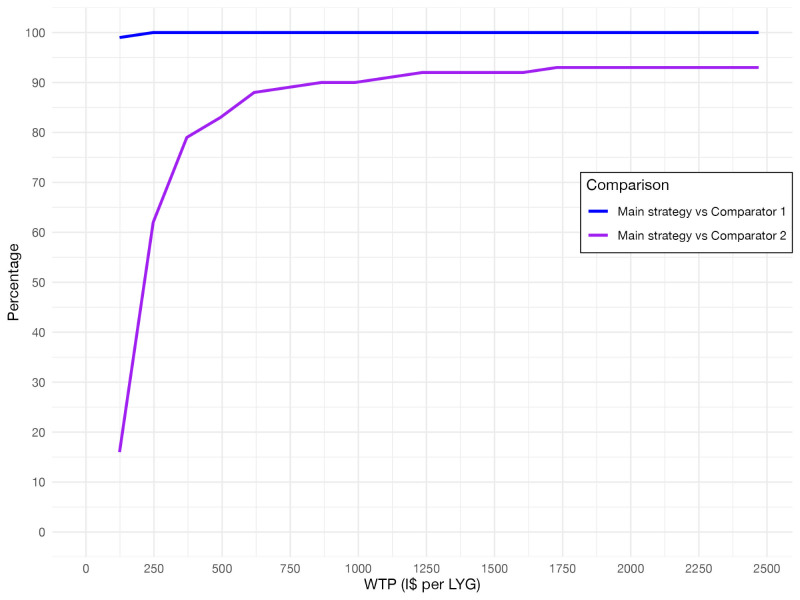
Probability of positive headroom results per WTP level. The lines represent the percentage of positive mean headroom results out of 100 simulations, with the Main strategy coverage and treatment adherence levels set at 60%. The same mean life years gained per woman and incremental costs per woman for 100 simulations were used to calculate the headroom for each of 100 simulations for a given WTP threshold. The calculation was repeated for the range of WTP thresholds. I$ – international dollar, LYG – life year gained, WP - willingness-to-pay.

The proportions of positive headroom results under 60% coverage and 60% adherence levels for both comparisons under a range of increasing WTP thresholds are presented (**Figure 2**). These are meant to represent the ‘acceptability’ of the evaluated strategy if the WTP were to change, which could be a result of increased funding for the health care system in general, greater priority given to funding cervical cancer screening, or other factors. This also demonstrates the uncertainty surrounding the WTP, particularly in the setting of Uganda, where an explicit threshold or a decision rule is not defined for evaluating health care interventions.

At these levels, the main strategy had 100% positive headroom vs comparator 1, starting from as low as 0.1 GDP per capita threshold (I$ 246.97 per life year gained) (**Figure 2**). In contrast, the proportion of positive headroom vs comparator 2 was much lower at the lower WTP levels, increasing rapidly between thresholds of 0.1 and 0.3 GDP per capita (I$ 246.97–740.91 per life year gained). 90% positivity was reached at approximately 0.35 GDP per capita per life year gained (I$ 864.40) in this comparison, which is still considerably lower than commonly used threshold levels. This illustrates how the interpretation of the strategy’s economic impact would change depending on the decision-making context.

### Headroom analysis: Maximum acceptable hrHPV test price

We also determined the mean headroom estimates of hrHPV test price variation (**Table 4**). A maximum price of I$ 30 per hrHPV was the threshold at which the main strategy maintained positive mean headroom vs comparator 1, with higher test prices leading to negative headroom. In contrast, the maximum acceptable test price was I$ 15 per test vs comparator 2.

**Table 4:** Headroom at different hrHPV test price levels in I$, presented as mean (SD)

	Price of hrHPV test*							
**Comparison**	**I$ 5**	**I$ 10**	**I$ 15**	**I$ 20**	**I$ 25**	**I$ 30**	**I$ 35**	**I$ 40**
**Main strategy vs comparator 1**	90.92 (21.14)	70.83 (21.52)	58.39 (20.02)	40.41 (21.95)	14.24 (16.94)	4.26 (19.12)	−21.01 (15.04)	−29.03 (17.53)
**Main strategy vs comparator 2**	44.07 (16.31)	23.83 (12.53)	8.45 (19.04)	−7.75 (21.7)	−27.31 (18.27)	−36.33 (24.34)	−63.22 (18.56)	−77.9 (20.49)

HrHPV – high-risk human papillomavirus, I$ – international dollar, SD – standard deviation

*The price of hrHPV test was varied from I$ 5 to I$ 40 for the self-sampled option and from I$ 10 to I$ 45 for the provider-collected option in I$ 5 steps. Coverage and adherence levels in the main strategy were fixed at 60%, as identified in the headroom analysis of minimum required levels. We ran 20 simulations for each hrHPV test price level. The mean headroom of 20 simulations for two comparisons is presented for each hrHPV test price level.

## DISCUSSION

We sought to identify the minimum required levels of screening coverage and adherence to treatment of precancerous lesions for the recommended hrHPV screen-and-treat strategy to be cost-effective in Uganda from a headroom analysis perspective. We found that for the hrHPV screen-and-treat strategy to be cost-effective in comparison with the existing VIA-based screen-and-treat strategy with low coverage (comparator 1, representing the current situation), a minimum of 30% screening coverage and a minimum of 30% adherence to treatment at each screening round are needed. When comparing the hrHPV-based strategy with a higher-coverage (i.e. 30%) VIA-based screening strategy with fewer screening (comparator 2), the hrHPV strategy became slightly less cost-effective, raising the minimum required levels of coverage and adherence to 60%. The cost of hrHPV tests is another important factor in these comparisons. At 60% coverage and 60% adherence, the maximum acceptable price per hrHPV test was I$ 30 when compared to VIA-based screening with low coverage, and I$ 15 when compared to VIA-based screening with higher coverage. While this early evaluation was not intended as a full cost-effectiveness assessment of hrHPV-based screening for Uganda, our findings are in agreement with previous economic evaluations, supporting it as a potentially cost-effective strategy for the country [30–32]. The differences in the modelled strategies, assumptions, and comparators make it difficult to directly compare our results. Specifically, due to the inclusion of programmatic costs of implementing the screening programme, as well as additional costs such as transportation, previously reported incremental cost-effectiveness ratio estimates of hrHPV-based screening are higher than those estimated by our model (**Online Supplementary Document**). We stress that our results should be interpreted only from the headroom perspective, representing room for spending on implementation of screening while remaining cost-effective, and not in terms of the incremental cost-effectiveness ratio. Given the uncertainty regarding the implementation parameters that motivated this early evaluation approach, collecting more reliable and context-specific data in Uganda is necessary for future full cost-effectiveness evaluations to inform policymakers.

### Coverage vs adherence: prioritisation

Our findings suggest that, from an economic perspective, higher levels of adherence to treatment of precancerous lesions could potentially be more valuable in this setting than higher screening coverage levels beyond a certain level. Compared to VIA-based screening with low coverage (i.e. 5%), increasing coverage of hrHPV-based strategy from 30% to 100% at 100% adherence increased the mean headroom by 70%, without meaningful increases after 60% coverage (**Table 2**). In contrast, a corresponding adherence increase at 100% coverage raised the mean headroom by 180%. In the other comparison, increasing coverage from 60% to 100% results only in a 4% headroom increase (**Table 3**), and a similar adherence increase produced a 3-fold headroom increase. Therefore, we suggest that interventions to achieve and maintain high treatment adherence should also be prioritised in this setting. Investing only in interventions to achieve high coverage and uptake of screening could potentially yield disappointing results. It is essential to target both coverage and adherence to ensure the strategy is cost-effective. Thorough consideration is needed for investments aimed at significantly increasing either parameter above certain levels to avoid wasting limited resources. In this context, countries like Uganda could benefit from implementation studies conducted alongside the early stages of the national rollout of the screening strategy. These studies can use various methods and be tailored to answer questions specific to the country’s needs.

The WHO targets of 70% coverage and 90% treatment adherence [33] could be seen as ambitious for LMICs, particularly at the early stages of adopting the strategy. Several demonstration projects in LMICs have reported reaching the adherence target in pilot implementations [34], but it is not clear how sustainable such a level would be in a national rollout in these settings in the long term. Projects in Uganda have also reported varying treatment adherence levels [35,36]. Based on our results, 30–60% treatment adherence could be considered as the minimum intermediate target for the strategy to be cost-effective in Uganda under a very strict WTP threshold of 0.1 GDP per capita per life year gained. Meanwhile, 60% coverage at each screening round, which corresponds to approximately 98% once-in-a-lifetime coverage, appeared sufficient to considerably reduce cervical cancer burden even compared to VIA-based screen-and-treat with 30% coverage, while leaving enough room to spend on the screening programme implementation. Further research is needed to identify the necessary context-specific interventions to ensure the sustainability of the recommended strategy in LMICs.

### HrHPV test price ceiling

At 60% coverage and 60% adherence levels, we identified I$ 30 as the maximum acceptable price per hrHPV test for Uganda when compared to VIA-based screening with low coverage (comparator 1). Compared to VIA-based screening with higher coverage (comparator 2), the maximum price resulting in positive mean headroom was I$ 15. With lower coverage and adherence levels, the acceptable price levels are also reduced. Therefore, the pricing of tests in this setting must be considered carefully to ensure that the strategy can be implemented cost-effectively.

The affordability of hrHPV tests has been described as a barrier to the implementation of hrHPV-based screening in LMICs [37]. Advancements in the cervical cancer test market are likely to increase availability and reduce costs in the future, but this will require continued cooperation between all stakeholders. While scaling up the rollout of hrHPV testing could result in lower prices due to higher purchase volumes, dropping to as low as EUR 5 (approx. I$ 3.15) per test [38], such volumes may not be realistic in low-resource settings during the early stages of implementation. Moreover, the rollout of the recommended strategy in Uganda will require substantial external funding. Annual governmental health expenditure in Uganda is estimated at only I$ 16 per capita, while external health expenditure, including resources from international donors, grant funding and other sources of financial aid, is at I$ 39 per capita [29]. Therefore, substantial reductions in hrHPV test pricing are needed for the recommended strategy to become more sustainable for the country’s health care system, and less reliant on external funders.

### Strengths and limitations

To our knowledge, this is the first study to evaluate the recommended hrHPV-based strategy from a headroom perspective for a low-resource setting. Instead of focussing on the cost-effectiveness of hrHPV testing, which has been generally established as part of analyses underpinning its adoption by the WHO as the recommended primary screening method worldwide, we focussed on both screening coverage and treatment adherence, as well as hrHPV test pricing, as the key parameters to ensure that the strategy could be cost-effective within strict resource constraints. Thus, our findings support the implementation of the WHO’s global elimination strategy in Uganda and in other LMICs.

There are several limitations to our study. First, we did not account for HPV vaccination policies or the prevalence of HPV vaccine immunity among the population. In Uganda, a two-dose HPV vaccination programme for adolescent girls was introduced in 2015 in some parts of the country, but uptake rates have been low [39,40]. For this reason, we did not expect that this would have a significant impact on our findings. We used life years gained instead of the more commonly used quality-adjusted life years as the health outcome measure. By offering the self-sampling option, hrHPV-based screening could result in utility gains from a health economic evaluation perspective, depending on measurement. Similarly, it could lead to utility loss due to higher rates of overtreatment, depending on assumptions about the utility decrements associated with screening and treatment procedures. Such assumptions have been shown to affect the cost-effectiveness outcomes in evaluations of cervical cancer screening [41]. However, the literature to inform such model parameters, particularly for LMICs, is limited, and therefore we chose not to include this measure. Furthermore, we modelled the general female population without women living with HIV in Uganda, where HIV prevalence is relatively high, estimated at 7.5% among females aged 15–49 [42]. HIV is known to increase the risk of HPV infection and cervical cancer progression and therefore would require a separate disease model. The cost-effectiveness of the strategy can be different among women living with HIV compared to the general population, requiring more frequent screenings and starting screening at an earlier age, as recommended by the WHO [12]. Finally, we did not include programmatic costs of hrHPV-based screening, in particular costs of interventions to increase and sustain coverage and adherence, so future evaluations should analyse these costs for this setting. It should also be noted that hrHPV test price estimates reported in the literature may not include additional costs, such as maintenance of the testing system or materials, which can be accounted for differently depending on the evaluation perspective. In general, we stress that the uncertainties regarding various inputs motivating our early evaluation approach limit the generalisability of the results.

## CONCLUSIONS

This early evaluation provides insights into potential minimum screening coverage and precancer treatment adherence targets for the hrHPV screen-and-treat strategy for Uganda to be cost-effective compared to the current VIA-based screen-and-treat strategies. If the strategy achieves at least 30% higher coverage per screening round compared to the existing VIA-based screening, with treatment adherence levels between 30–60% and the hrHPV tests priced below I$ 30 per test, it is likely to be cost-effective in this setting. Further research is needed to provide more country-specific data to inform future economic evaluation models supporting decision-making regarding the implementation of hrHPV-based screening in Uganda.

## Additional material


Online Supplementary Document
