# Supplementary material for: Early evaluation of a screen-and-treat strategy using high-risk HPV testing for Uganda: Implications for screening coverage and treatment
Source: J Glob Health. 2024 Sep 20;14:04157. doi: 10.7189/jogh.14.04157 (PMC11414519; doi:10.7189/jogh.14.04157)
Supplement: Online Supplementary Document [file jogh-14-04157-s001.pdf]

Online Supplementary Document for "Early evaluation of a screen-and-treat strategy using high-risk human papillomavirus testing for Uganda: implications for screening coverage and treatment adherence"

Marat Sultanov[1], Jurjen van der Schans[1,2], Jaap AR Koot[1], Marcel JW Greuter[3,] Janine de Zeeuw[1], Carolyn Nakisige[4], Jogchum J Beltman[5], Marlieke de Fouw[5], Jelle Stekelenburg[1,6], Geertruida H de Bock[7]

[1]Global Health Unit, Department of Health Sciences, University Medical Center Groningen, University of Groningen, Groningen, Netherlands
[2]Department of Economics, Econometrics and Finance, Faculty of Economics and Business, University of Groningen, Groningen, Netherlands
[3]Department of Radiology, University Medical Center Groningen, University of Groningen, Groningen, Netherlands
[4]Uganda Cancer Institute, Kampala, Uganda
[5]Department of Gynecology, Leiden University Medical Center, Leiden University, Leiden, Netherlands
[6]Department of Obstetrics and Gynecology, Medical Center Leeuwarden, Leeuwarden, Netherlands
[7]Department of Epidemiology, University Medical Center Groningen, University of Groningen, Netherlands

Disease model structure

The state transition model included 7 mutually exclusive states: high-risk human papillomavirus (hrHPV)-negative, hrHPV-positive, cervical intraepithelial neoplasia (CIN) 1, CIN 2, CIN 3, Cancer, and Dead. The annual transition probabilities retained from the original model [1] are presented in Figure S1. In addition, all-cause (background) mortality probabilities (i.e. transition probabilities to Dead state from all states) were age-specific and determined using country-specific female mortality data [2].

Figure S1. Disease model structure

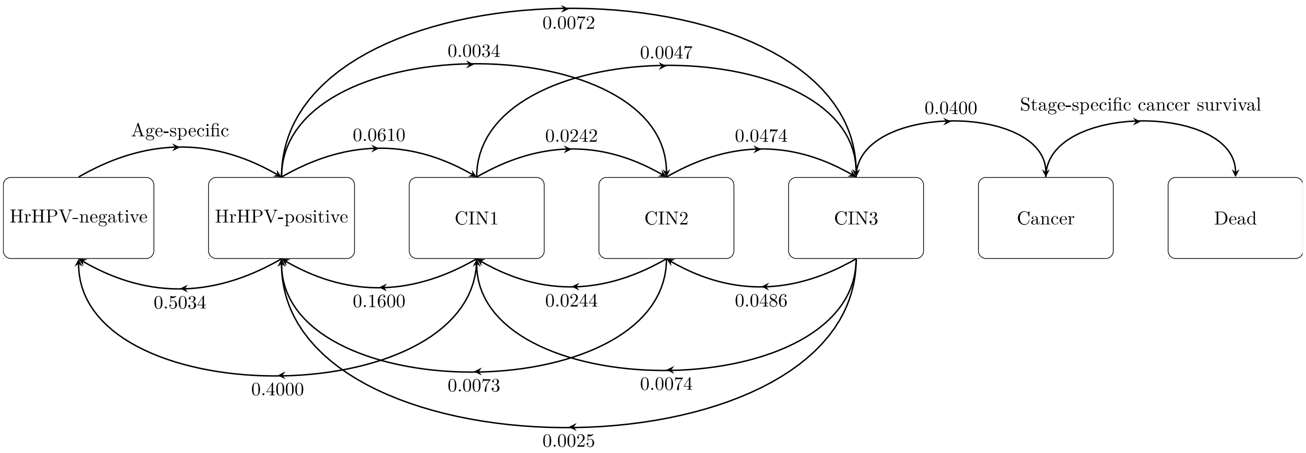

The key assumptions involved in the disease model are as follows:

- Progression to any CIN1, CIN2 or CIN3 occurs only from hrHPV-positive state;
- Progression to cervical cancer occurs only from CIN3 state;
- There is no direct regression from CIN2 or CIN3 to hrHPV-negative;
- Cervical cancer is modeled as a single state, with each woman entering the state assigned a cancer stage (according to International Federation of Gynecology and Obstetrics (FIGO) system), based on reported stage distribution, and cancer survival based on reported survival rates.

HrHPV prevalence adjustment

The following reported prevalence rates were used as reference targets for adjusting the transition probability from hrHPV-negative to hrHPV-positive state [3]:

- Ages 25-34: 0.31
- Ages 35-44: 0.25
- Ages 45-54: 0.22
- Ages 55-60: 0.31

The age-specific prevalence rates of hrHPV (defined as proportion of women in the hrHPV-positive state out of all women alive at a given cycle (equivalent to age)) using adjusted transition probabilities (Table S1) are presented in Figure S2. Assuming the reference prevalence rates are uniformly distributed within each age group, the prevalence rates produced using adjusted transition probabilities were considered sufficiently close.

Table S1. Adjusted age-specific transition probabilities used in the model

Age range	Annual transition probability: from hrHPV-negative to hrHPV-positive
15-19	0.40
20-24	0.45
25-29	0.35
30-34	0.25
35-39	0.20
40-44	0.15
45-49	0.18
50-54	0.25
55-59	0.23
60-64	0.18
65-69	0.15

Figure S2. Age-specific hrHPV prevalence using adjusted transition probabilities

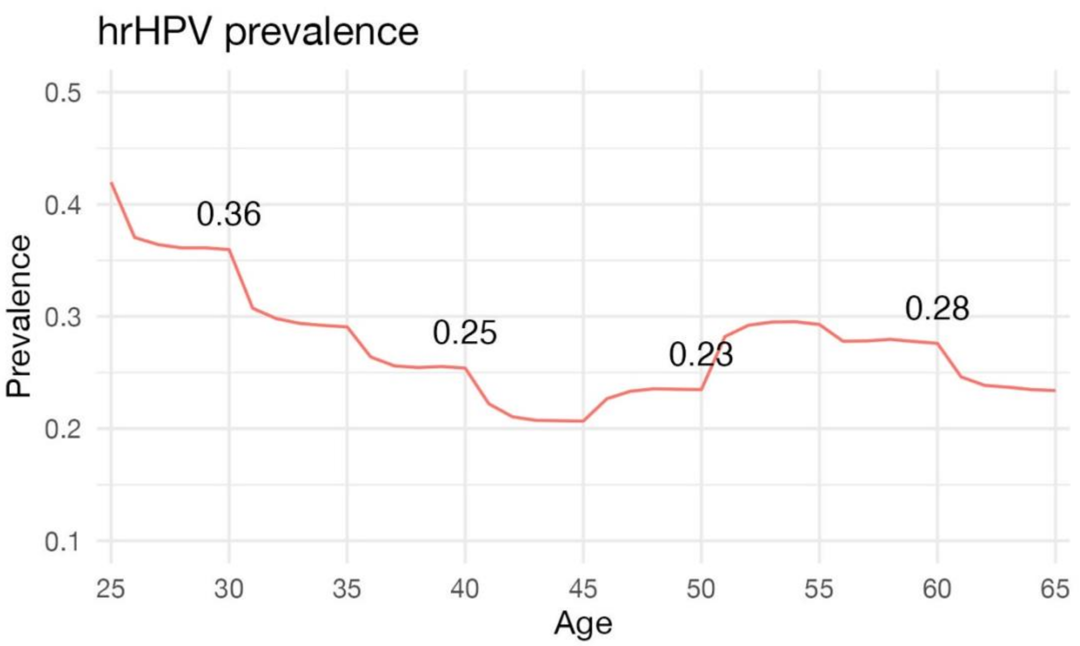

Prevalence was calculated as proportion of women in hrHPV-positive model state out of all women alive at each model cycle. Since all women in the model start at age 0, cycle numbers are equivalent to ages.

Model parameters

A targeted literature search was conducted to identify relevant sources to inform parameters for the Uganda setting. Conducting a full systematic review to inform model parameters was not feasible and out of scope of this early evaluation, considering the limited literature available for this setting. The full list of parameters and corresponding sources and assumptions is presented in Table S2. Various sources needed to be combined to identify the best available evidence for the model parameters, including data from a WHO multi-country African demonstration project, previous evaluation of hrHPV screening in Uganda, multi-country African cancer registry study and relevant trials from other Sub-Saharan countries.

Table S2. Full list of parameters

Parameter	Value	Source
**Transitions between health states**		
Transition probabilities between hrHPV-positive, CIN1, CIN2, CIN3 and Cancer states	Unchanged from original model (Supplementary Figure 1)	
Age-specific transition probabilities from hrHPV-negative to hrHPV-positive	Adjusted to reported prevalence rates [3] (Supplementary Figure 2, Supplementary Table 1)	

**Screening coverage and pre-cancer treatment adherence parameters**			
Proportion of women choosing self-sampling hrHPV test option	83.2%		[4]
Proportion of women choosing provider-collected hrHPV test option	26.8%		
Screening coverage per screening round	Main strategy	100% (Effectiveness gap analysis)	-
		10-100% (Headroom analysis)	
	Comparator 1	5%	Assumption
	Comparator 2	30%	Assumption
Proportion of women returning hrHPV self-sample	95%		Assumption
Adherence to ablative treatment	Effectiveness gap analysis	84% – all strategies	[5]
	Headroom analysis of coverage/adherence levels	10-100% - Main strategy	-
Adherence to excisional treatment	Effectiveness gap analysis	84% – all strategies	[5]
	Headroom analysis of coverage/adherence levels	10-100% - Main strategy	-
**Screening test and precancer treatment parameters**			
HrHPV test, self-sampled	Sensitivity	92.9%	[6]
	Specificity	93.9%	
HrHPV test, provider-collected	Sensitivity	96.4%	
	Specificity	94.2%	
Proportion of women eligible for ablative treatment	87.7%		[7]
VIA test (Comparator 1 and 2)	Sensitivity	82.4%	[8]
	Specificity	87.4%	
Ablative treatment efficacy	74.6%		[9]
Excisional treatment efficacy	83.6%		
**Costs (health system perspective, 2022 international dollars)**			
VIA test (Comparator 1 and 2), VIA assessment (Main strategy)	7.84		[10]
Ablative treatment (cryotherapy)	36.48		[10]
Excisional treatment (LEEP)	377.32		[10]
hrHPV test, self-sampled	Effectiveness gap analysis, Headroom analysis of coverage/adherence levels	15	Assumption
	Headroom analysis of hrHPV test price	5-40	
hrHPV test, provider-collected	Effectiveness gap analysis, Headroom analysis of coverage/adherence levels	20	Assumption
	Headroom analysis of hrHPV test price	10-45	
Cancer treatment (single cost per cancer case)	FIGO stages I-II	2401.20	[11]
	FIGO stages III-IV	3179.97	

Gross Domestic Product per capita	2467.9			[12]
**Other**				
All-cause age-specific mortality	Global Health Observatory data – indicator nqx (probability of dying between ages x and x+n)			[2]
**Cancer survival**				
Cancer stage distribution	Stage I	11.405%		[13]
	Stage II	19.505%		
	Stage III	45.005%		
	Stage IV	20.805%		
Cervical cancer survival per stage	Stages I-II		Year 1: 0.83	[14]
			Year 2: 0.72	
			Year 3: 0.61	
			Year 4: 0.55	
			Year 5: 0.5	
	Stages III-IV		Year 1: 0.53	
			Year 2: 0.48	
			Year 3: 0.3	
			Year 4: 0.26	
			Year 5: 0.23	

Screening flows

Figure S3. Screening flow – Main strategy (hrHPV screen-and-treat)

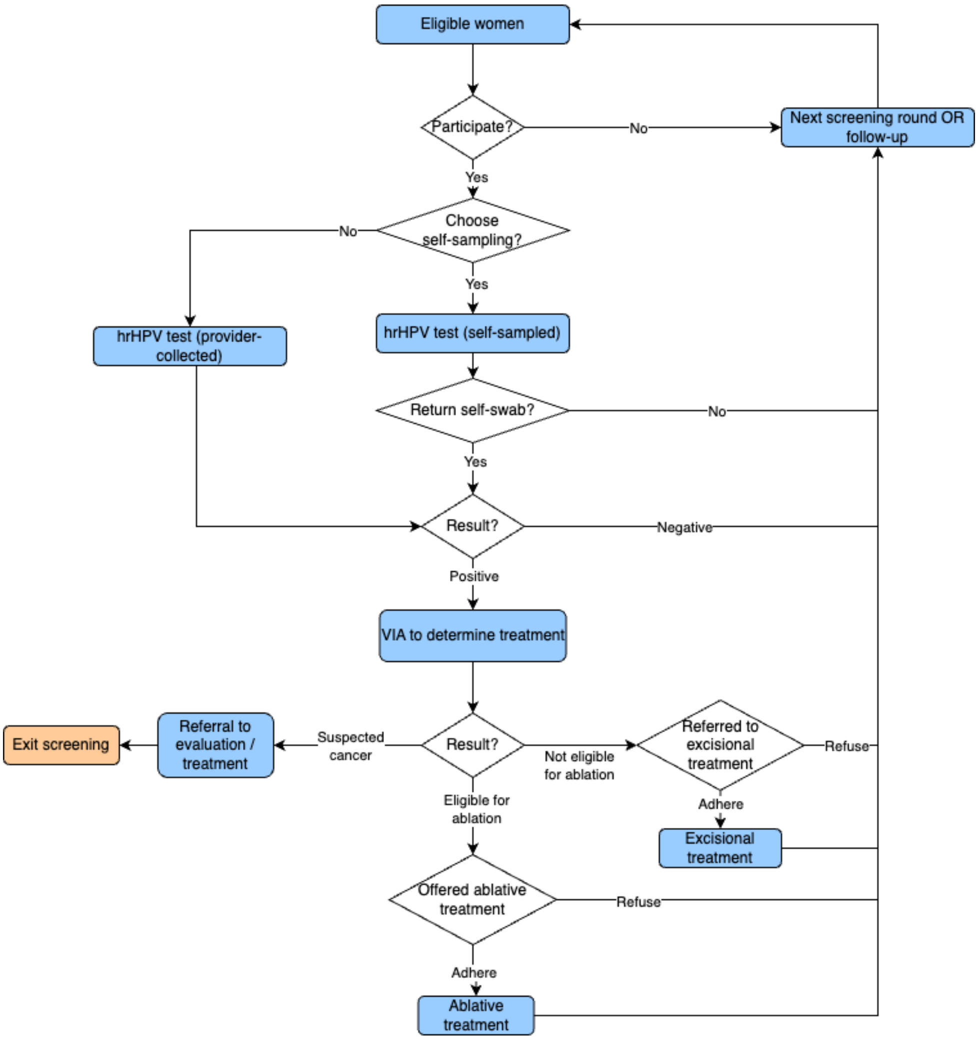

The screening flow is a simplified representation of the WHO recommended screen-and-treat strategy with hrHPV testing evaluated in this study. The "Participate?" step represents coverage per screening round parameter as defined in this study. The "Adhere" option following "Offered ablative treatment" and "Referred to excisional treatment" represents adherence to precancer treatment. Women in Cancer state are always identified as "Suspected cancer" at the VIA assessment step and exit the screening flow.

Figure S4. Screening flow - Comparator 1 and 2 (VIA screen-and-treat)

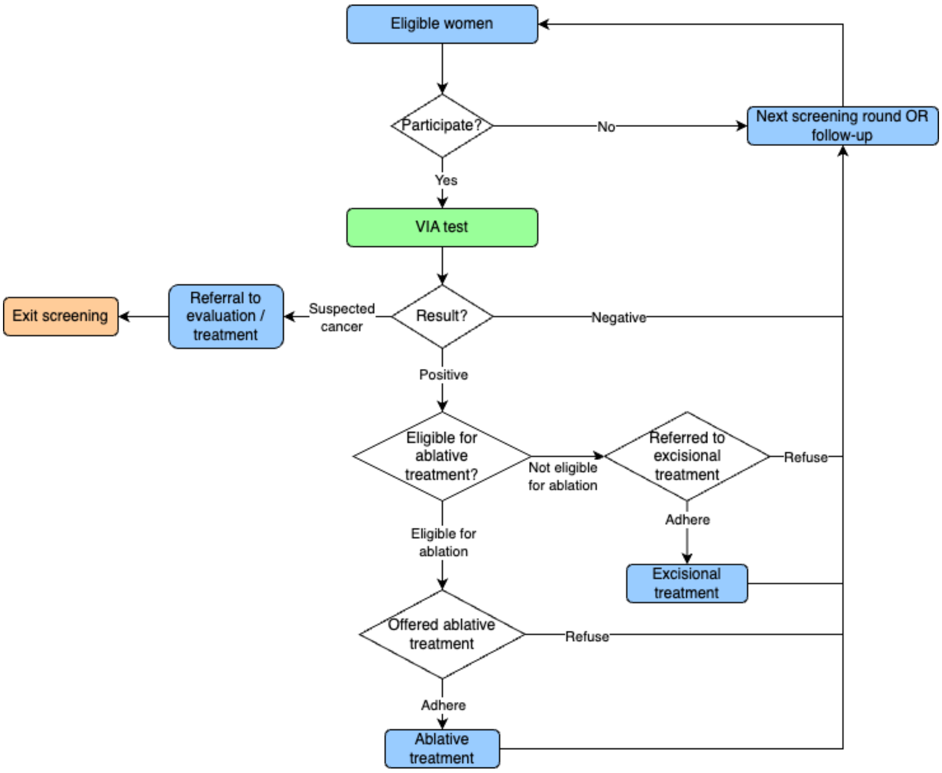

The screening flow is a simplified representation of the VIA-based screen-and-treat strategy as the current screening policy in Uganda. Same treatment options as in the Main strategy are present. The difference from the Main strategy is in the primary screening test (VIA), eligibility and intervals between screening (Table 1 of main manuscript text). The "Participate?" step represents coverage per screening round as defined in this study. The "Adhere" option following "Offered ablative treatment" and "Referred to excisional treatment" represents adherence to precancer treatment parameter. Women in Cancer state are always identified as "Suspected cancer" at the VIA assessment step and exit the screening flow.

Results - Life years gained

Table S3. Life years gained - Main strategy versus Comparator 1 (mean (SD))

		**Adherence to pre-cancer treatment (%)**									
		100	90	80	70	60	50	40	30	20	10
**Screening coverage (%)**	100	0.43 (0.07)	0.41 (0.09)	0.39 (0.07)	0.4 (0.08)	0.37 (0.09)	0.36 (0.09)	0.33 (0.09)	0.3 (0.07)	0.23 (0.09)	0.15 (0.09)
	90	0.39 (0.07)	0.4 (0.1)	0.36 (0.09)	0.38 (0.08)	0.37 (0.07)	0.35 (0.09)	0.31 (0.08)	0.28 (0.06)	0.2 (0.08)	0.09 (0.09)
	80	0.36 (0.09)	0.37 (0.08)	0.34 (0.07)	0.35 (0.06)	0.33 (0.06)	0.3 (0.06)	0.24 (0.07)	0.25 (0.09)	0.2 (0.07)	0.11 (0.08)
	70	0.32 (0.06)	0.32 (0.08)	0.34 (0.06)	0.31 (0.09)	0.28 (0.1)	0.24 (0.08)	0.25 (0.1)	0.2 (0.11)	0.15 (0.09)	0.07 (0.09)
	60	0.32 (0.06)	0.28 (0.06)	0.28 (0.07)	0.3 (0.06)	0.26 (0.07)	0.25 (0.08)	0.2 (0.07)	0.17 (0.07)	0.11 (0.09)	0.05 (0.07)
	50	0.24 (0.09)	0.23 (0.07)	0.23 (0.11)	0.24 (0.08)	0.2 (0.07)	0.19 (0.09)	0.15 (0.06)	0.08 (0.08)	0.07 (0.09)	0.03 (0.08)

	40	0.21 (0.08)	0.2 (0.09)	0.21 (0.08)	0.19 (0.09)	0.15 (0.09)	0.16 (0.08)	0.14 (0.09)	0.11 (0.08)	0.04 (0.08)	0.01 (0.07)
	30	0.17 (0.09)	0.15 (0.08)	0.15 (0.09)	0.12 (0.08)	0.12 (0.08)	0.08 (0.1)	0.05 (0.08)	0.05 (0.08)	0.06 (0.1)	0 (0.08)
	20	0.08 (0.09)	0.07 (0.08)	0.11 (0.08)	0.07 (0.1)	0.05 (0.07)	0.04 (0.1)	0.05 (0.07)	0.03 (0.08)	-0.04 (0.1)	0 (0.07)
	10	0.05 (0.08)	0.02 (0.09)	0 (0.06)	0.02 (0.08)	-0.01 (0.08)	-0.02 (0.09)	-0.04 (0.08)	-0.06 (0.08)	-0.03 (0.09)	-0.04 (0.09)

For each coverage and adherence level (100 combinations), 20 simulations of all strategies were run. Mean life years gained per woman produced by Main strategy versus Comparator 1, out of 20 simulations for each combination of coverage and adherence levels, are presented.

Table S4. Life years gained - Main strategy versus Comparator 2 (mean (SD))

		**Adherence to pre-cancer treatment (%)**									
		100	90	80	70	60	50	40	30	20	10
**Screening coverage (%)**	100	0.29 (0.06)	0.28 (0.08)	0.25 (0.06)	0.27 (0.08)	0.23 (0.08)	0.21 (0.07)	0.19 (0.08)	0.18 (0.1)	0.1 (0.07)	0 (0.07)
	90	0.26 (0.08)	0.28 (0.11)	0.22 (0.09)	0.23 (0.09)	0.22 (0.08)	0.23 (0.06)	0.19 (0.07)	0.11 (0.09)	0.07 (0.07)	-0.02 (0.09)
	80	0.23 (0.07)	0.26 (0.08)	0.23 (0.1)	0.2 (0.06)	0.18 (0.09)	0.16 (0.1)	0.13 (0.1)	0.11 (0.1)	0.08 (0.1)	-0.03 (0.1)
	70	0.19 (0.09)	0.18 (0.09)	0.21 (0.08)	0.16 (0.06)	0.16 (0.09)	0.15 (0.09)	0.13 (0.09)	0.07 (0.09)	0.02 (0.09)	-0.09 (0.09)
	60	0.18 (0.06)	0.12 (0.08)	0.16 (0.09)	0.15 (0.08)	0.13 (0.08)	0.11 (0.07)	0.08 (0.09)	0.03 (0.06)	-0.01 (0.1)	-0.09 (0.08)
	50	0.12 (0.09)	0.12 (0.08)	0.11 (0.1)	0.1 (0.08)	0.07 (0.06)	0.05 (0.09)	0.02 (0.07)	-0.02 (0.1)	-0.06 (0.09)	-0.12 (0.06)
	40	0.1 (0.07)	0.06 (0.07)	0.07 (0.1)	0.05 (0.1)	0.01 (0.09)	0.01 (0.08)	0.03 (0.07)	-0.07 (0.08)	-0.11 (0.08)	-0.1 (0.08)
	30	0 (0.09)	0.02 (0.08)	0.01 (0.09)	-0.01 (0.1)	-0.01 (0.09)	-0.05 (0.1)	-0.08 (0.09)	-0.06 (0.08)	-0.12 (0.07)	-0.13 (0.09)
	20	-0.06 (0.09)	-0.06 (0.08)	0.02 (0.08)	-0.07 (0.09)	-0.09 (0.09)	-0.09 (0.08)	-0.1 (0.1)	-0.12 (0.09)	-0.17 (0.09)	-0.17 (0.08)
	10	-0.11 (0.07)	-0.14 (0.1)	-0.12 (0.08)	-0.1 (0.09)	-0.14 (0.1)	-0.16 (0.08)	-0.19 (0.1)	-0.2 (0.09)	-0.17 (0.09)	-0.19 (0.1)

For each coverage and adherence level (100 combinations), 20 simulations of all strategies were run. Mean life years gained per woman produced by Main strategy versus Comparator, out of 20 simulations for each combination of coverage and adherence levels, are presented

Context - previous evaluations

Mezei, et al [16] estimated the incremental cost-effectiveness ratio of I$470 per life year gained for a strategy with 5 screening rounds, 70% coverage and adherence of 78.6%, compared to no screening. In our analysis, the comparable 20% screening coverage per screening round (leading to 67% lifetime screening coverage) and 80% treatment adherence level produced a cost-effectiveness ratio of - I$40.33 per life year gained compared to no screening. This result suggests that the strategy was simultaneously cost-saving and gaining additional life years in our model, making it a “dominant” strategy compared to no screening.

This big difference in the cost-effectiveness outcome is most likely due to inclusion of programmatic costs of screening by Mezei, et al., which leads to much higher incremental costs of the screening strategy in their analysis, as well as higher cancer treatment cost assumptions and transition probability to cervical cancer used in our model, which lead to more costs accumulated without screening. As noted in the manuscript, without modeling programmatic costs of screening, our results should only be interpreted in the context of headroom estimates representing the potential amount left to spend for programmatic costs while remaining cost-effective, and not in terms of incremental cost-effectiveness ratio. Moreover, we did not apply discounting, which also results in more favorable estimates of cost-effectiveness.

Campos, et al [17] estimated the incremental cost-effectiveness of screening three times in a lifetime with 70% coverage at I$350 per life year gained compared to a baseline 30% one-time coverage screening in Uganda. In our analysis, 60% coverage (corresponding to lifetime 68% three-times coverage) and 70% adherence resulted in a cost-effectiveness ratio of I$26.55 per life year gained. While some of cost parameters for our model were derived from their study, Campos, et al. also included costs related to transportation and women's time in their analysis, which may have contributed to the substantial difference between our cost-effectiveness estimates. Their study also reported additional 0.064 life years gained with each 10% increase in coverage. In our analysis, a comparable increase in coverage per screening round from 60% to 70% (corresponding to an increase from 68.26% to 83.69% three-time coverage over 5 screening rounds) similarly resulted in additional 0.056 life years gained compared to current VIA-based screening with 5% coverage, suggesting that while our model outcomes in terms of the saving life years may be broadly comparable, different cost assumptions result in the discrepancy in cost-effectiveness estimates.

References

1. Castañeda M. Molecular Testing as Triage in Organized Cervical Cancer Screening: Early Economic Evaluation Using Headroom Analysis (submitted for publication). 2023.

2. World Health Organization. Global Health Observatory Data Repository. 2023. https://www.who.int/data/gho. Accessed 7 Apr 2023.

3. Jeronimo J, Bansil P, Lim J, Peck R, Paul P, Amador JJ, et al. A multicountry evaluation of careHPV testing, visual inspection with acetic acid, and papanicolaou testing for the detection of cervical cancer. Int J Gynecol Cancer Off J Int Gynecol Cancer Soc. 2014;24:576–85.

4. Modibbo F, Iregbu KC, Okuma J, Leeman A, Kasius A, de Koning M, et al. Randomized trial evaluating self-sampling for HPV DNA based tests for cervical cancer screening in Nigeria. Infect Agent Cancer. 2017;12:11.

5. Yang L, Boily M-C, Rönn MM, Obiri-Yeboah D, Morhason-Bello I, Meda N, et al. Regional and country-level trends in cervical cancer screening coverage in sub-Saharan Africa: A systematic analysis of population-based surveys (2000–2020). PLOS Med. 2023;20:e1004143.

6. Polman NJ, Ebisch RMF, Heideman DAM, Melchers WJG, Bekkers RLM, Molijn AC, et al. Performance of human papillomavirus testing on self-collected versus clinician-collected samples for the detection of cervical intraepithelial neoplasia of grade 2 or worse: a randomised, paired screen-positive, non-inferiority trial. Lancet Oncol. 2019;20:229–38.

7. World Health Organization. Prevention of cervical cancer through screening using visual inspection with acetic acid (VIA) and treatment with cryotherapy. A demonstration project in six African countries. 2012.

8. Fokom-Domgue J, Combescure C, Fokom-Defo V, Tebeu PM, Vassilakos P, Kengne AP, et al. Performance of alternative strategies for primary cervical cancer screening in sub-Saharan Africa: systematic review and meta-analysis of diagnostic test accuracy studies. BMJ. 2015;351:h3084.

9. Zhang L, Sauvaget C, Mosquera I, Basu P. Efficacy, acceptability and safety of ablative versus excisional procedure in the treatment of histologically confirmed CIN2/3: A systematic review. BJOG Int J Obstet Gynaecol. 2023;130:153–61.

10. Campos NG, Tsu V, Jeronimo J, Mvundura M, Lee K, Kim JJ. When and how often to screen for cervical cancer in three low- and middle-income countries: A cost-effectiveness analysis. Papillomavirus Res. 2015;1:38–58.

11. Campos NG, Tsu V, Jeronimo J, Njama-Meya D, Mvundura M, Kim JJ. Cost-effectiveness of an HPV self-collection campaign in Uganda: comparing models for delivery of cervical cancer screening in a low-income setting. Health Policy Plan. 2017;32:956–68.

12. World Bank. World Bank Open Data. https://data.worldbank.org/. Accessed 19 Jan 2022.

13. Mwaka AD, Garimoi CO, Were EM, Roland M, Wabinga H, Lyratzopoulos G. Social, demographic and healthcare factors associated with stage at diagnosis of cervical cancer: cross-sectional study in a tertiary hospital in Northern Uganda. BMJ Open. 2016;6:e007690.

14. Sengayi-Muchengeti M, Joko-Fru WY, Miranda-Filho A, Egue M, Akele-Akpo M-T, N'da G, et al. Cervical cancer survival in sub-Saharan Africa by age, stage at diagnosis and Human Development Index: A population-based registry study. Int J Cancer. 2020;147:3037–48.

15. Griesel M, Seraphin TP, Mezger NCS, Hämmerl L, Feuchtner J, Joko-Fru WY, et al. Cervical Cancer in Sub-Saharan Africa: A Multinational Population-Based Cohort Study of Care and Guideline Adherence. The Oncologist. 2021;26:e807–16.

16. Mezei AK, Pedersen HN, Sy S, Regan C, Mitchell-Foster SM, Byamugisha J, et al. Community-based HPV self-collection versus visual inspection with acetic acid in Uganda: a cost-effectiveness analysis of the ASPIRE trial. BMJ Open. 2018;8:e020484.

17. Campos NG, Tsu V, Jeronimo J, Mvundura M, Lee K, Kim JJ. To expand coverage, or increase frequency: Quantifying the tradeoffs between equity and efficiency facing cervical cancer screening programs in low-resource settings. Int J Cancer. 2017;140:1293–305.
